# CS27109, A Selective Thyroid Hormone Receptor-*β* Agonist Alleviates Metabolic-Associated Fatty Liver Disease in Murine Models

**DOI:** 10.1155/2023/4950597

**Published:** 2023-02-14

**Authors:** Shengjian Huang, Zhou Deng, Wei Wang, Guoqiang Liao, Yiru Zhao, Hua Zhong, Qian Zhang, Jing Liu, Xuhua Mao, Beizhong Chen, Desi Pan, You Zhou

**Affiliations:** ^1^Shenzhen Chipscreen Biosciences Co., Ltd., Shenzhen 518052, China; ^2^Chengdu Chipscreen Pharmaceutical Ltd., Chengdu 610213, China

## Abstract

**Background/Aim:**

Thyroid hormone receptor-*β* (THR-*β*) agonists play crucial roles in dyslipidemia and metabolic associated fatty liver disease (MAFLD). We developed a novel oral and liver-targeted THR-*β* agonist, CS27109, and evaluated its efficacy in the treatment of metabolic disorders.

**Materials and Methods:**

We evaluated *in vitro* and *in vivo* efficacy and/or safety of CS27109 along with MGL3196 (a phase III THR-*β* agonist).

**Results:**

CS27109 showed pronounced activity and selectivity to THR-*β* and favorable PK properties, which was equivalent to MGL3196. In the hamster model, animals treated with a high dose of CS27109 showed equivalent reductions in serum TC and LDL-c with groups treated with MGL3196. In the rat model, CS27109 and MGL3196 reduced serum ALT, TC, TG, LDL-c, liver weight ratio, and liver steatosis. CS27109 simultaneously decreased liver TG and TC, and MGL3196 additionally reduced AST. In the mouse model, CS27109 dose-dependently reduced serum AST, ALT, liver inflammation, and NAS score, and also downregulated TC, LDL-c, liver steatosis, and fibrosis, but not in a dose-dependent manner. MGL3196 revealed an equivalent effect with CS27109 in that model. CS27109 also exhibited tolerable toxicity to the heart.

**Conclusions:**

CS27109 shows comparative *in vitro* and *in vivo* efficacy with MGL3196, suggesting its potential therapeutic application in the treatment of MAFLD such as dyslipidemia and steatohepatitis.

## 1. Introduction

Metabolic-associated fatty liver disease (MAFLD), a nomenclature, was recently proposed by experts to integrate the accuracy of understanding patient heterogeneity with the so-called nonalcoholic fatty liver disease (NAFLD) [1–3]. This terminology reflects a heterogeneous phenotype caused by the pathological accumulation of triglycerides and other lipids in hepatocytes, leading to steatohepatitis, liver fibrosis, cirrhosis, and eventually hepatocellular carcinoma. MAFLD is also associated with obesity, dyslipidemia, type 2 diabetes mellitus (T2DM), and insulin resistance [4–6]. Exercise and diet control are considered effective ways to prevent MAFLD, but no drugs have been approved to treat this disease, especially in patients who develop severe steatohepatitis and liver fibrosis [7]. Drugs targeting glycolipid metabolism alone or with other mechanistic candidates have been emerging as potential strategies to overcome chronic disease, one of these drugs is thyroid hormone receptor-*β* (THR-*β*) agonists [8–10].

Thyroid hormones (THs) are secreted by the thyroid and play a role in mediating growth, development, and metabolism [11, 12]. The thyroid hormone receptor (THR) forms a complex with TH in a ligand-dependent way to regulate gene expression downstream [13]. Dysfunction or abnormity of this complex results in a variety of diseases, including especially obesity and metabolic disorders [14, 15]. Two THR isoforms are reported, thyroid hormone receptor-*α* (THR-*α*) and thyroid hormone receptor-*β* (THR-*β*), in which THR-*α* is expressed mainly in the brain and heart and THR-*β* is highly expressed in the liver [16, 17]. In terms of MAFLD, the TH/THR axis regulates liver triglyceride and cholesterol metabolism via enhancing lipid mobilization and increasing free fatty acid transportation [18]. However, the endogenous active TH form, 3,5,3′-triiodothyronine (T3), triggers both THRs simultaneously, and thyromimetics without selectivity display side effects on the heart, muscle, and bone due to the activation of THR-*α* [19, 20]. Therefore, developing THR-*β* agonists with high potency and specificity is a potential strategy to target MAFLD with minimal side effects.

Several THR-*β* agonists with oral bioavailability and liver-targeted properties have been developed and showed preclinical efficacy in NAFLD and nonalcoholic steatohepatitis (NASH) models [21–26]. Recently, two THR-*β* agonists, MGL3196 (Resmetirom) and VK2809, have progressed to phase III clinical trials for the treatment of NASH and hyperlipidemia [19, 27]. VK2809 is a precursor of KB-141 that was terminated due to side effects such as increased heart rate, manifesting a reduction in serum and liver TG without affecting heart function. MGL3196 is an oral and liver-targeted THR-*β* agonist with pronounced activity and selectivity towards THR-*β*. According to the randomized, double-blind, phase 3b MAESTRO-NAFLD-1 trial, Resmetirom has the potential to be the first approved drug for patients with NASH. In addition, both drugs also show efficacy in alleviating lipid disorder and NASH in preclinical studies [28, 29]. However, diarrhea and increased bowel movements are the main side effects observed in patients receiving Resmetirom. Therefore, we still need to develop new compounds with higher selectivity to THR-*β* and eliminate side effects.

Consequently, we designed and developed a new compound named CS27109 with a brand new molecular structure. This compound exhibited considerable *in vitro* activity and selectivity to THR-*β*, similar to MGL3196. In this study, we focus on the *in vivo* efficacy of CS27109 in three different animal models to test its potential for treating MAFLD along with MGL3196. Each animal model represents different aspects of MAFLD. Our results indicate that CS27109 showed comparative efficacy with MGL3196 in these models, suggesting that it is a potential candidate to target MAFLD such as hypercholesterolemia and steatohepatitis.

## 2. Materials and Methods

### 2.1. Compounds

Both CS27109 and MGL3196 were synthesized at Shenzhen Chipscreen Biosciences Co., Ltd. with over 99% purity. Compounds were dissolved in sterile DMSO and 0.2 Carboxymethylcellulose sodium (CMC-Na) and 0.1% Tween 80 (solvent) for *in vitro* and *in vivo* studies, respectively. T3 was purchased from MedChemExpress (Hycultec GmbH, Beutelsbach, Germany) and dissolved in sterile DMSO for *in vitro* testing.

### 2.2. Luciferase Reporter Gene Assays

We applied a luciferase reporter gene assay to assess the capacity of drugs to bind and activate THR*α* or THR*β*. First, we seeded 5 × 10^3^ CV-1 cells for each well into 96-well plates and incubated for 24 hours at 37°C and 5% CO_2_. We then transiently transfected mixed THR-*α* or THR-*β* expression plasmids (with DR4) into liposomes with a firefly luciferase reporter (2 : 2 : 1, THR*α* : DR4 : GFP or 3 : 3 : 1, THR*β* : DR4 : GFP) and then mixed with the X-tremeGENE HP DNA Transfection Reagent (Roche, Basel, BS, Switzerland). After 24-hour incubation, CS27109 (0.01–30 M), MGL-3196 (0.01–30 M), and T3 (0.000001–1 M) were added to the transfected cells for 22 hours. We then removed the medium and added 60 *μ*l of 1x Cell Culture Lysis and 5x reagent (Promega, E153A) to each well. We then transferred 50 *μ*l of lysate into a 96-well white plate and measured on a Tecan microplate reader (Männedorf, Switzerland) to determine the GFP fluorescence value (Ex485/Em520). Finally, we added 30 *μ*l of Luciferase Assay Buffer (Promega, E151A) to each well to detect Luci values. 50% activation (AC_50_) for each compound was calculated based on these values.

### 2.3. hERG Test

hERG test was performed by Ice Bioscience Co., Ltd. (Beijing, China) using the QPatch HTX method according to the manufacturer's instruction. 5 doses of CS27109 were tested, including 0.3, 1, 3, 10, and 10 mm, each one with a duplicate. The tested values (current) were normalized to the negative control (0.1% DMSO), and IC_50_ was calculated based on the normalized data.

### 2.4. Pharmacokinetic Studies

PK in the mouse: 6 C57/B6 mice ♂ aged 6–7 weeks were evenly divided into two groups and administered 10 mg/kg CS27109 or MGL3196 once by oral gavage, respectively. Blood was collected for each mouse 0.5, 1, 2, 3, 4, 6, 8, 24, and 30 hours (H) after compound administration. Then, the serum was isolated and an extractant (containing 200 ng/ml methanol) was added to the serum. Samples were assessed by liquid chromatography-tandem mass spectrometry (LC-MS/MS) according to the standard procedure.

PK in the rat: 8 SD rats ♂ aged 5–6 weeks were evenly divided into two groups and administered 10 mg/kg CS27109 or MGL3196 once by oral gavage, respectively. Blood was collected for each mouse 0.5, 1, 2, 4, 6, 8, 10, and 24H after compound administration. Then, the serum was isolated, and an extractant (containing 200 ng/ml methanol) was added to the serum. Samples were assessed by liquid chromatography-tandem mass spectrometry (LC-MS/MS) according to the standard procedure.

Tissue distribution: 12 SD rats ♂ aged 5–6 weeks were evenly divided into two groups and administered 10 mg/kg CS27109 or MGL3196 once by oral gavage, respectively. Half of mice for each group (*n* = 3) were sacrificed 2H and 8H after drug administration, respectively. Blood, heart, and liver were collected at each time point. Blood was processed and detected as mentioned previously. The heart and liver were homogenized and added extractant. The protein of tissue samples was precipitated by vortex oscillation, and the supernatant was collected and determined by LC-MS/MS procedures.

### 2.5. Animal Studies

**Figure 1:**
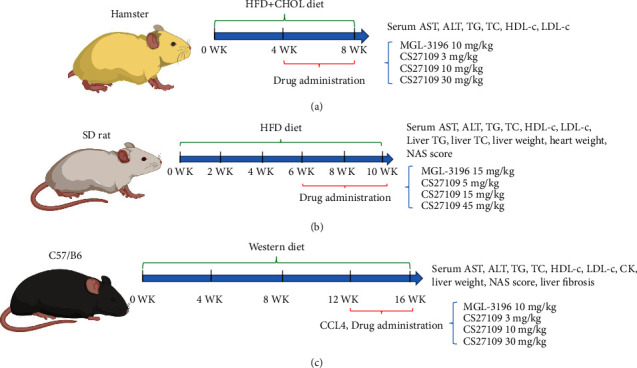
Schematic representation of *in vivo* studies: (a) experimental procedure of developing a hamster model with HFD + CHOL and testing drug efficacy, (b) experimental procedure of developing an SD rat model with HFD and testing drug efficacy, and (c) experimental procedure of developing a mouse model with WD + CCL4 and testing drug efficacy.

All animal studies were carried out adhering to the Guiding Principles in the Care and Use of Animals approved by the Council of Chengdu Chipscreen Pharmaceutical Ltd. All animals were housed in our animal facility (temperature, 20–25°C; humidity, 40–70%; 12/12-hour light/dark cycle). Schematic representation of animal studies was illustrated in Figure 1.

Hamster study: 58 golden hamsters ♂ aged 6–8 weeks were purchased from Beijing Vital River Laboratory Animal Technology Co., Ltd. 10 animals were kept with a diet of food control as normal control, and the other 48 animals were fed 40% fat and 1.25% cholesterol (HFD-CHOL, D12108C, Research diet) as a model group. After 4 weeks of feeding, the animals in the model group were randomly assigned to 5 subgroups based on the serum TC level: model (*n* = 10), MGL3196 10 mg/kg (*n* = 10), CS27109 3 mg/kg (*n* = 10), CS27109 10 mg/kg (*n* = 9), and CS27109 30 mg/kg (*n* = 9). Animals in the normal and model group were given solvent by oral gavage. All drugs, including solvent, were administered consecutively for 4 weeks and once a day. After sacrifice, blood was collected and serum was isolated for the AST, ALT, TG, TC, LDL-c, and HDL-c test.

SD rat study: 58 SD rats ♂ aged 5–6 weeks were purchased from Beijing Huafukang Biotechnology Co., Ltd. 9 animals were kept with a food control diet as normal control, and the other 49 animals were fed 40% fat, 1.25% cholesterol and 0.5% sodium cholate (HFD, D12109C, SYSE BIO, Changzhou, China) as a model group. After 6 weeks of feeding, the animals in the model group were randomly assigned to 5 subgroups based on the serum TC level: model (*n* = 9), MGL3196 15 mg/kg (*n* = 10), CS27109 5 mg/kg (*n* = 10), CS27109 15 mg/kg (*n* = 10), and CS27109 45 mg/kg (*n* = 10) (Figure 1(b)). Animals in the normal and model groups were given solvent by oral gavage. All drugs, including solvent, were administered consecutively for 4 weeks and once a day. After sacrifice, blood was collected and serum was isolated for the AST, ALT, TG, TC, LDL-c, and HDL-c test, and livers and hearts were weighed to calculate the liver weight ratio and the heart weight ratio, respectively. The right lobe of the liver of each rat was harvested for the TG and TC test (A110-1-1 and A111-1-1, Nanjing Jiancheng Institute of Bioengineering, Nanjing, China) according to the manufacturer's instructions. The left lobe of the liver was collected for hematoxylin-eosin staining (H and E staining) to quantify liver steatosis, inflammation, and ballooning.

C57/B6 mouse study: 61 C57/B6 mice ♂ aged 6–7 weeks were purchased from Beijing Vital River Laboratory Animal Technology Co., Ltd. Six animals were kept with a chow diet as normal control, and the other 55 animals were fed 60% fat diet (Western diet, D12492, Research diet) as the model group. After 12 weeks of feeding, the animals in the model group were administered 1 ml/kg of carbon tetrachloride (CCL4, 1 : 4 dissolved in olive oil) by intraperitoneal injection three times a week (4 weeks in total) and simultaneously randomly assigned to 5 subgroups: model (*n* = 11), MGL3196 10 mg/kg (*n* = 11), CS27109 3 mg/kg (*n* = 11), CS27109 10 mg/kg (*n* = 10), and CS27109 30 mg/kg (*n* = 12) (Figure 1(c)). Animals in the normal and model groups were given solvent by oral gavage. All drugs, including solvent, were administered consecutively for 4 weeks and once a day. After sacrifice, blood was collected and serum was isolated for the AST, ALT, TG, TC, LDL-c, HDL-c, and CK tests, and the livers were weighed to calculate the liver weight ratio. The left lobe of the liver of each mouse was collected for hematoxylin-eosin staining (H and E staining) and sirius red staining.

### 2.6. Pathological Studies of the Liver

The liver samples were fixed in 10% formalin and embedded in paraffin using standard methods. The 4 *μ*m paraffin sections were then assessed for liver histology by H and E staining and liver fibrosis by sirius red staining. The stained sections were panoramic scanned with a NanoZoomer Digital Pathology scanner (S210, Hamamatsu, Japan), and then observed with different visual fields at different magnifications, and scored for different liver lobes in the sections. The total NAS score represents the sum of the scores for steatosis (0–3), lobular inflammation (0–3), and ballooning (0–2), and ranges from 0–8 [30]. VIS7.0 software was used to analyze the area of liver fibrosis in the panoramic scanning SR staining sections, and the percentage of liver fibrosis area in the whole section area was calculated.

### 2.7. Statistical Analysis

Data are expressed as the mean ± SD and the GraphPad Prism 8.0 software (GraphPad Software Inc, CA, USA) were used for statistical analysis. *One-way analysis of variance (ANOVA)* was used for comparison between groups. *The Bonferroni* test was used for data with homogeneous variance, and *the Dunnett* test was used for data with uneven variance. *p* < 0.05 indicates that the difference is statistically significant.

## 3. Results

### 3.1. CS27109 is a Potent THR-*β* Agonist

**Figure 2:**
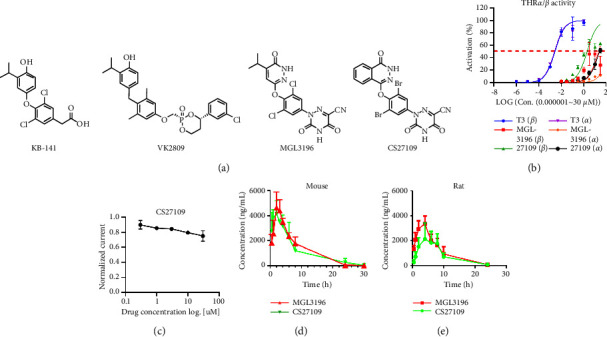
Chemical structure, THR-*β* activity, and PK properties of CS27109: (a) chemical structure of several oral and liver-targeted THR-*β* agonists, including KB-141, VK2809, MGL3196, and CS27109, (b) THR-*α* and THR-*β* activity for each compound were determined by a luciferase reporter gene assay, (c) hERG test of CS27109 was conducted by the QPatch HTX, (d) mouse, and (e) rat PK studies for CS27109 and MGL3196.

Unlike VK-2809 and KB-141, MGL-3196 and CS27109 gain a better selectivity by changing the phosphoric acid group or carboxyl group to 3,5-dioxo-2,3,4,5-tetrahydro-1,2,4-triazine-6-carbonitrile (Figure 2(a)). The replacement of 4-isopropylpyridine-3(2H)-one of MGL3196 to phthalazin-1(2H)-one of CS27109 might increase the potency of THR-*β* activation. To test THR-*β* activity and selectivity, we conducted a luciferase reporter gene assay. Results showed that CS27109 was potent to activate THR-*β* with lower AC_50_ compared with MGL3196 (1.98 *μ*M vs. 21.32 *μ*M, Figure 2(b) and Table 1). However, CS27109 also activated THR-*α* with lower AC_50_ compared with MGL3196 (25.09 *μ*M vs. 192.1 *μ*M, Figure 2(b) and Table 1). We then determined THR-*α*/THR-*β* selectivity for both compounds compared with T3, an agonist with unbiased selectivity (0.89). The selectivity for CS27109 and MGL3196 was 12.67 and 9.01, respectively (Table 1). Furthermore, the hERG test indicated that CS27109 was cardiac tolerable (Figure 2(c)). These data suggest that CS27109 has more potent activity and equivalent selectivity towards THR-*β* compared with MGL3196 and shows pronounced cardiac safety.

### 3.2. CS27109 has Favorable PK Properties

We then conducted PK studies of CS27109 and MGL3196 in both mouse and rat at 10 mg/kg. Results showed that comparative PK properties were observed in mouse between CS27109 and MGL3196 (Figure 2(d)), while MGL3196 exhibited slightly higher PK properties in rat compared with CS27109 (Figure 2(e)). We further characterized the tissue distribution of both drugs in the rat. Results showed that the pattern of tissue distribution between the two compounds was different, which drug concentration was increased for CS27109 and decreased for MGL3196 from 2H to 8H in three tested organs (Table 2). The average of concentration liver/heart for MGL3196 and CS27109 was 22.325 and 26.69, respectively, indicating both drugs were liver-targeted.

MGL3196 was studied in several preclinical models, in which 10 mg/kg was an effective dose frequently used in mouse models. Therefore, we chose this dose for MGL3196 as a positive control and for CS27109 as a medium dose in mouse studies. We then expanded doses (range 3–30 mg/kg) to check a dose-response pattern for CS27109. The AUC of CS27109 in mouse was 1.5-fold higher than that in rat, so we selected a dose range from 5 to 45 mg/kg in the rat model study.

### 3.3. CS27109 Improves Dyslipidemia in the HFD-CHOL Hamster Model

*In vitro* and PK results suggest that CS27109 should exhibit pharmacodynamical efficacy in animal studies. To achieve this, we established three different animals to test the efficacy of both compounds. The first is an HFD-CHOL hamster model fed a high-fat and high-cholesterol diet for 8 weeks in total (Figure 1(a)). This model was characterized by an increase in serum TG, TC, and LDL-c level (Table 3), which resembles hyperlipidemia and hypercholesterolemia in human patients. In addition, serum AST and ALT were also elevated in this model compared with the normal control, indicating a possible liver injury (Table 3).

After 4-week treatments, CS27109 dose-dependently reduced serum TC and LDL-c with a significant effect in the medium and high-dose groups but not in the low-dose group (Table 3). Similarly, MGL3196 10 mg/kg also lowered the serum TC and LDL-c level and showed an equivalent effect with high-dose CS27109 (30 mg/kg). Additionally, CS27109 reduced serum AST in a dose-dependent manner but only with a significant effect in the high-dose group (Table 3). Interestingly, none of the CS27109 group exhibited serum TG reduction even with a dose-dependent trend, which is inferior to MGL3196. Neither CS27109 nor MGL3196 decreased serum ALT (Table 3). However, low- and high-dose CS27109 was also found to reduce serum HDL-c, which is still in the normal psychological range. Collectively, CS27109 and MGL3196 potentiate the amelioration of dyslipidemia, especially in the regulation of serum TC and LDL-c.

### 3.4. CS27109 Improves Lipid Metabolism and Liver Steatosis in HFD Rat Model

The SD rat with HFD was reported to be a suitable model for the study of dyslipidemia and liver steatosis study [31]. In our study, we found that serum TC was dramatically elevated in rats even after two weeks of HFD feeding (data not shown). This study lasts 10 weeks in total, in which the duration of drug administration is 4 weeks (Figure 1(b)). Compared with the control of the chow diet (the normal group), the animals in the model group demonstrated an extremely increased serum TC and LDL-c, but not TG and HDL-c (Table 4), which is consistent with hypercholesterolemia in human disease. Furthermore, liver TC but not TG was also elevated in the model group, according to the serum result. Furthermore, serum AST and ALT also increased dramatically in the model group, indicating liver injury in this model (Table 4). The enlarged liver is also a characteristic of this model, resulting in a higher liver weight ratio compared with the normal control (Table 4).

After 4-week drug administration, three doses of CS27109 significantly reduced serum ALT, TG, TC, and LDL-c (Table 4), in which TC and LDL-c were dose-dependent. Compared with MGL3196, CS27109 exhibited a comparative reduction in serum TC and LDL-c at the same dosage. However, MGL3196 reduced serum AST and TG more than for the three groups of CS27109. In accordance with the serum test, CS27109 also reduced liver TC and TG content, while MGL3196 did not exert a significant effect on neither index (Table 4). Furthermore, CS27109 as well as MGL3196 dramatically reduced the liver weight ratio. Only CS27109 30 mg/kg increased the heart weight ratio but was still close to the normal group, suggesting that neither CS27109 nor MGL3196 adversely affected cardiac function in this model (Table 4).

**Figure 3:**
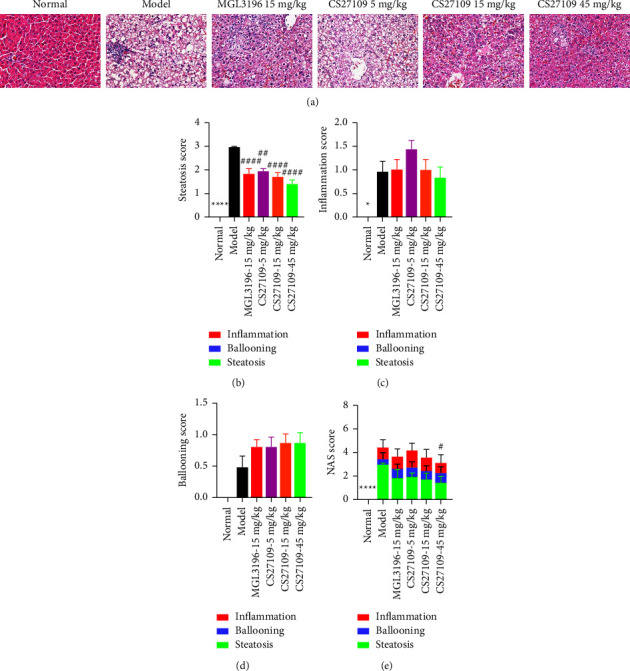
CS27109 lessens liver steatosis in HFD rat model: (a) H and E staining of livers among different treating groups after 4-week drug intervention, (b) steatosis, (c) inflammation, (d) ballooning, and (e) NAS scores of liver pathology among different groups. ^*∗*^and # represents normal vs. model and drugs vs. model, respectively. ^*∗*^*p* < 0.05, ^*∗∗∗∗*^*p* < 0.0001, ^#^*p* < 0.05, ^##^*p* < 0.01, and ^####^*p* < 0.0001.

Subsequently, we assessed liver pathology by H and E staining among different groups of treatments (Figure 3(a)). As shown, animals in the model group exhibited higher liver steatosis, inflammation, and ballooning than the normal group, especially liver steatosis (Figures 3(b)–3(d)). The NAS score reached 5, which is characterized as a mild MASH model (Figure 3(e)). As expected, both CS27109 and MGL3196 lessened steatosis, but not inflammation and ballooning scores, in which CS27109 showed a dose-dependent reduction in steatosis. However, both drugs slightly but not significantly increased the ballooning score, implying possible liver toxicity for both drugs, which needs to be elucidated in the further study. CS27109 also dose-dependently reduced liver inflammation, but without a significant effect compared with the model group. Statistically, only a high dose of CS27109 decreased the NAS score due to its robust efficacy in regulating liver steatosis (Figure 3(e)). However, MGL3196 exhibited a tendency to reduce the NAS score, but without significance.

### 3.5. CS27109 Attenuates Liver Inflammation and Prevents Fibrosis in the WD + CCL4 Mouse Model

Most murine models with HFD even after long-term feeding feature as steatohepatitis, but rarely liver fibrosis. Chemical reagents such as CCL4 can induce liver injury and fibrosis in different murine models [26]. Furthermore, animals that received WD and CCL4 simultaneously are similar to the corresponding human disease. To validate this model, we fed C57/B6 male mice with WD for 12 weeks and then injected CCL4 three times a week for 4 weeks (Figure 1(c)). After sacrifice, mice in the model group showed a tremendous increase in serum ALT and AST (Table 5) compared with the normal control. Furthermore, serum TC and LDL-c were also significantly elevated, but serum HDL-c was reduced in the model group (Table 5). However, serum TG and CK level were not affected between the two groups. Sadly, the liver weight ratio did not increase in this model (Table 5), which was different from the HFD only models.

After 4-week drug administration, medium and high doses CS27109, as well as MGL3196 dramatically reduced serum ALT and AST, in which CS27109 showed a dose-dependent pattern (Table 5). Serum TC and LDL-c were also significantly reduced in 4 drug-treated groups compared with the model group, close to the normal group (Table 5). Neither drug affected serum TG, CK, or liver weight ratio.

**Figure 4:**
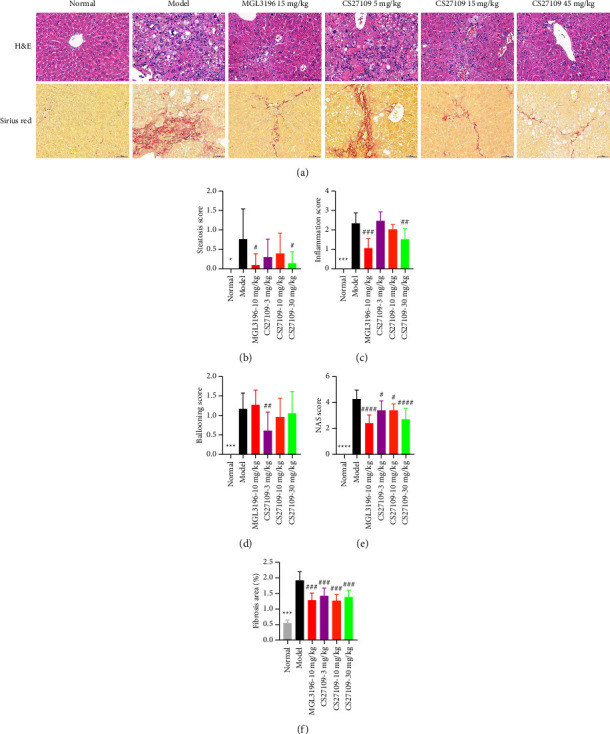
CS27109 mitigates steatohepatitis and hepatic fibrosis in WD + CCL4 mouse model: (a) H and E and sirius red staining of livers among different treating groups after 4-week drug intervention for determining steatohepatitis and fibrosis, respectively, (b) steatosis, (c) inflammation, (d) ballooning (e) NAS score, and (f) fibrosis area of liver pathology among different groups. ^*∗*^ and ^#^ represents normal vs. model and drugs vs. model, respectively. ^*∗*^*p* < 0.05, ^*∗∗∗*^*p* < 0.001, ^*∗∗∗∗*^*p* < 0.0001, ^#^*p* < 0.05, ^##^*p* < 0.01, ^###^*p* < 0.001, and ^####^*p* < 0.0001.

We further examined hepatitis and liver fibrosis of this model by pathological studies (Figure 4(a)). According to the reported studies, our model also displayed severe liver inflammation (Figure 4(c)) and fibrosis (Figure 4(f)) with mild steatosis and ballooning (Figures 4(b) and 4(d)). After drug intervention, we observed a notable reduction in liver steatosis and inflammation in MGL3196 and CS27109 high-dose groups as compared with the model group (Figures 4(b) and 4(c)). However, only the low-dose group CS27109 significantly reduced liver ballooning (Figure 4(d)). Furthermore, the NAS score which combines scoring of liver steatosis, inflammation, and ballooning was dramatically decreased in all drug-treated groups, in which the high-dosed CS27019 group showed equivalent efficacy with the MGL3196 treated group (Figure 4(e)). Furthermore, three doses of CS27109 and MGL3196 also prominently reduced the area of the fibrosis in relation to the model group (Figure 4(f)). Collectively, we suggest both compounds are capable to attenuate liver inflammation and prevent fibrosis.

## 4. Discussion

The NAFLD was coined by Ludwig to distinguish it from alcoholic fatty liver disease (AFLD) characterized by excessive drug intake [32]. This terminology has been used for decades, but its accuracy has been challenged due to its complexity and heterogeneity, which impede clinical management and pathogenic understanding. To solve this problem, MAFLD was proposed to better reflect current knowledge of the disease, and a consensus was reached in 2020 by an international expert panel. This new definition links liver disease closer to metabolic disorders and simplifies the diagnostic process used by NAFLD [33]. In other words, MAFLD emphasizes the role of metabolic dysfunction in it instead of exclusion of significant alcohol intake or other chronic liver diseases. This renaming will further increase the prevalence of MAFLD and prompt disease awareness, research, and clinical management. Therefore, we urge researchers to use this MAFLD nomenclature instead of NAFLD or NASH which is still popular and used in other articles.

As MAFLD was recently proposed, there is few research on the development of animal models, particularly under this terminology, but tons of studies were found based on NAFLD or NASH [34, 35]. Therefore, we refer to the NAFLD or NASH animal models, as they also share some common features with MAFLD. We focus on the metabolic and hepatic disorders of our drug, and key characteristics of MAFLD. To achieve this goal, we chose three different animal models representing different aspects of the disease: dyslipidemia, steatohepatitis, and fibrosis. The golden hamster model with HFD-CHOL illustrates the human disease of hypertriglyceridemia and hypercholesterolemia, in which serum TG and TC along with LDL-c are tremendously elevated. Serum AST and ALT are also dramatically increased, indicating that liver injury in this model may be due to lipotoxicity through excessive lipid intake. The hamster was reported to be more similar to human lipid metabolism than other rodents such as rat and mouse [36]. To develop a model with both dyslipidemia and steatohepatitis that frequently occur in humans, we fed SD rats with longer term (10 weeks) and an extra 0.5% cholate in HFD. Together with cholesterol, cholate was reported to induce the progressive development of liver steatosis and inflammation in 6–24 weeks [37]. We confirm this observation in our study, demonstrating elevated serum TC and LDL-c, pronounced liver steatosis, mild inflammation, and ballooning. However, HFD alone rarely induced liver fibrosis in murine models even with a longer time (more than 20 weeks). In order to shorten research time, chemicals such as CCL4 and streptozotocin (STZ) are often introduced into the HFD system to accelerate liver injury and fibrosis [35]. In a previous study, WD + CCL4 resulted in stage 3 fibrosis at 12 weeks and hepatocellular carcinoma development at 24 weeks in mice [38]. Intriguingly, these models shared dysregulated molecular pathways and immunologic characteristics closely with the human disease by transcriptomic analysis. We verified this model according to the literature with similar observations. In detail, our model shows an extraordinary increase in serum AST and ALT, indicating a severe liver injury. This abnormality is confirmed by the pathological study, demonstrating high scores of liver inflammation, ballooning, and fibrosis. However, lipoproteins such as TC and LDL-c in the blood are only significantly increased, but the fold changes are not comparable with those in the hamster and rat models, which may be due to the toxicity of CCL4, which restricts food intake and lipogenesis.

Lifestyle interventions such as diet control and exercise are the main strategies for the treatment of patients with MAFLD, but most patients with advanced stage of the disease unwillingly implement such changes [39]. Therefore, pharmacological interventions remain highly demanded. Dozens of targets are reported to play different roles in MAFLD progression, in which THR-*β* mediates cholesterol metabolism and excretion through bile acids. Moreover, THR-*β* agonists alleviate liver injury including inflammation and fibrosis by reducing lipotoxicity and liver fat. Safety and efficacy are two aspects of an ideal drug candidate, where MGL3196, the first phase III THR-*β* agonist, already showed promising efficacy and tolerable safety. To compete with MGL3196, we designed CS27109, a new compound with high potency and selectivity towards THR-*β*, and tested *in vitro* and *in vivo* efficacy of both compounds.

In general, CS27109 shows equivalent efficacy with MGL3196 in improving features of MAFLD such as dyslipidemia and steatohepatitis. By further analysis, CS27109 only dose-dependently reduces serum TC and LDL-c in all animal models, which is in line with its mechanism of cholesterol regulation [39]. Some other parameters are significantly modulated by CS27109 in one murine model, but not in others. For example, CS27109 medium and high doses reduce serum ALT in the rat and mouse model, but not in the hamster model. Another example is that the high dose of CS27109 reduces the liver inflammation score in the mouse but not in the rat model, even showing a tendency in it. These data indicate that more information needs to be combined to determine drug efficacy. Compared with MGL3196, CS27109 showed an equivalent reduction in serum TC and LDL-c, but an inferior reduction in serum TG, suggesting its potential for hypercholesterolemia. In the WD + CCL4 model, simultaneously intervention with CCL4 and compounds suggests that CS27109 potentially prevents progression from liver steatohepatitis to fibrosis, whether its therapeutic effect in this model is still undefined. In our another study (manuscript is submitting to another journal), we evaluated combination therapy between CS27109 and CS17919 (a ASK1 inhibitor developed by our team) in a similar model in which CCL4 induction was started at week 8 (8 weeks in total) and drug intervention began 4-week after CCL4 induction. In this study, we found CS27109 alone was sufficient to reverse liver fibrosis and achieve a synergistic effect with CS17919. Therefore, CS27109 might also hold potential for the treatment of metabolic-related fibrosis.

Drug safety is considered as same important as efficacy, where cardiac disorder is the main side effect of THR-*β* agonists. We hypothesize that CS27109 has an equivalent safety profile to MGL3196 which has been confirmed in clinical trials. We validate our hypothesis as followings: first, CS27109 and MGL3196 share a similar chemical structure, indicating that both compounds might also share similar safety attributes; second, the hERG result implies that CS27109 has a minimum cardiac effect (IC_50_ > 30 *μ*M); third, PK and tissue distribution reveals that CS27109 rarely activate THR-*α* until extremely high dose; fourth, CS27109 rarely affects serum CK and cardiac weight ratio, two key parameters related with cardiac function in two separate models. Furthermore, animals treated with MGL3196 exhibit diarrhea, similar to clinical observation, but is rarely observed in rodents that received CS27109. Collectively, we suggest that CS27109 is a potent THR-*β* agonist with acceptable tolerability.

## 5. Conclusion

In summary, we designed a novel THR-*β* agonist CS27109 with potent *in vitro* activity and selectivity. CS27109 also showed favorable PK properties and was liver-targeted. Furthermore, CS27109 exhibited equivalent serum reduction in TC and LDL-c, mitigation of steatohepatitis, and prevention of liver fibrosis with MGL3196. These findings indicate that CS27109 plays a potential role in treating of MAFLD.

## Figures and Tables

**Table 1:** AC_50_ of THR-*α* and THR-*β* and selectivity of THR-*α*/THR-*β* for CS27109 and MGL3196.

Compound		CS27109	MGL3196	T3
AC_50_ (*μ*M)	*β*	1.980	21.32	0.002780
	*α*	25.09	192.1	0.002481

Selectivity (*α*/*β*)		12.67	9.01	0.89

**Table 2:** Tissue distribution of MGL3196 and CS27109 in SD rat.

Compound	MGL3196		CS27109	
	2H	8H	2H	8H
Serum (ng/mL)	1403.6	506.1	670	3624.4
Heart (ng/g)	155.6	128.2	95.7	309
Liver (ng/g)	3851.4	2551.3	1666.5	11116.4

**Table 3:** Biochemical test in HFD-CHOL hamster model after 4-week drug intervention. ^*∗*^ and ^#^represents normal vs. model and drugs vs. model, respectively. ^*∗∗*^*p* < 0.01, ^*∗∗∗*^*p* < 0.001, ^#^*p* < 0.05, and ^###^*p* < 0.001.

Groups	Normal	Model	MGL-3196	CS27109-L	CS27109-M	CS27109-H
AST (U/L)	98 ± 45^*∗∗∗*^	638 ± 323	752 ± 261	564 ± 317	582 ± 383	416 ± 158
ALT (U/L)	94 ± 58^*∗∗*^	278 ± 174	206 ± 47	226 ± 104	141 ± 133	127 ± 47^#^
TG (mmol/L)	2.1 ± 1.1^*∗∗*^	26 ± 24	5.9 ± 5.6^#^	32 ± 31	17 ± 26	11 ± 19
TC (mmol/L)	3.1 ± 0.72^*∗∗∗*^	37 ± 13	18 ± 6.3^###^	29 ± 8.6	25 ± 7.4^#^	19 ± 7.9^###^
LDL-c (mmol/L)	1.3 ± 0.54^*∗∗∗*^	17 ± 6.1	8.6 ± 3.3^###^	13 ± 4.3	12 ± 3.8^#^	8.7 ± 4.1^###^
HDL-c (mmol/L)	1.6 ± 0.45^*∗∗∗*^	3.6 ± 1.1	2.9 ± 0.36	2.6 ± 0.62^#^	3.0 ± 0.52	2.6 ± 0.53^#^

**Table 4:** Biochemical, organ/body weight ratio, and liver TC and TG test in HFD SD rat model after 4-week drug intervention. ^*∗*^ and ^#^ represents normal vs. model and drugs vs. model, respectively. ^*∗∗*^*p* < 0.01, ^*∗∗∗*^*p* < 0.001, ^*∗∗∗∗*^*p* < 0.0001, ^#^*p* < 0.05, ^##^*p* < 0.01, ^###^*p* < 0.001, and ^####^*p* < 0.0001. L/B (%) stands for liver/body weight ratio and H/B stands for heart/body weight ratio.

Groups	Normal	Model	MGL-3196	CS27109-L	CS27109-M	CS27109-H
AST (U/L)	103 ± 21^*∗∗∗*^	365 ± 212	185 ± 66^#^	213 ± 78	218 ± 82	253 ± 131
ALT (U/L)	32 ± 6.8^*∗∗∗∗*^	166 ± 95	74 ± 24^##^	85 ± 34^##^	68 ± 24^##^	89 ± 50^#^
TG (mmol/L)	0.53 ± 0.34	0.82 ± 0.73	0.11 ± 0.03^*∗∗∗*^	0.19 ± 0.08^##^	0.22 ± 0.06^##^	0.20 ± 0.08^##^
TC (mmol/L)	1.5 ± 0.20^*∗∗∗∗*^	20 ± 13	3.3 ± 1.3^####^	4.4 ± 1.8^####^	2.9 ± 0.98^####^	2.3 ± 0.65^####^
LDL-c (mmol/L)	0.28 ± 0.06^*∗∗∗∗*^	9.7 ± 6.1	1.1 ± 0.65^####^	1.6 ± 0.74^####^	0.84 ± 0.44^####^	0.59 ± 0.32^####^
HDL-c (mmol/L)	1.1 ± 0.13	1.2 ± 0.44	0.94 ± 0.26	0.78 ± 0.18^#^	0.98 ± 0.32	1.0 ± 0.30
L/B (%)	2.8 ± 0.26^*∗∗∗∗*^	5.1 ± 0.75	3.7 ± 0.42^####^	4.0 ± 0.52^###^	4.1 ± 0.56^##^	3.9 ± 0.54^###^
H/B (%)	0.28 ± 0.02^*∗∗*^	0.24 ± 0.02	0.27 ± 0.02	0.27 ± 0.03	0.28 ± 0.02^#^	0.27 ± 0.02
Liver TG (*μ*mol/g)	0.75 ± 12	102 ± 36	113 ± 38	71 ± 28	54 ± 24^#^	69 ± 21
Liver TC (*μ*mol/g)	6.0 ± 0.76^*∗∗∗∗*^	13 ± 2.2	12 ± 1.7	11 ± 1.1	10 ± 0.74^##^	10 ± 2.2^##^

**Table 5:** Biochemical and liver/body weight ratio test in WD + CCL4 model after 4-week drug intervention. ^*∗*^ and ^#^ represents normal vs. model and drugs vs. model, respectively. ^*∗∗*^*p* < 0.01, ^*∗∗∗*^*p* < 0.001, ^#^*p* < 0.05, ^##^*p* < 0.01, and ^###^*p* < 0.001. L/B (%) stands for liver/body weight ratio.

Groups	Normal	Model	MGL-3196	CS27109-L	CS27109-M	CS27109-H
AST (U/L)	116 ± 71^*∗∗∗*^	10245 ± 4955	2178 ± 769^###^	13447 ± 6633	4804 ± 1121^#^	3660 ± 1208^##^
ALT (U/L)	54 ± 15^*∗∗∗*^	14003 ± 4205	4658 ± 1639^###^	19162 ± 4012	10478 ± 2131^##^	7832 ± 2422^###^
TG (mmol/L)	0.91 ± 0.14	1.2 ± 0.34	1.0 ± 0.23	1.2 ± 0.34	1.2 ± 0.19	1.2 ± 0.18
TC (mmol/L)	3.0 ± 0.10^*∗∗*^	4.1 ± 0.97	1.9 ± 0.17^###^	2.3 ± 0.40^###^	2.2 ± 0.36^###^	2.4 ± 0.48^###^
LDL-c (mmol/L)	0.43 ± 0.04^*∗∗∗*^	1.1 ± 0.35	0.33 ± 0.05^###^	0.40 ± 0.10^###^	0.37 ± 0.07^###^	0.39 ± 0.11^###^
HDL-c (mmol/L)	2.0 ± 0.08^*∗∗*^	1.5 ± 0.25	1.2 ± 0.13^#^	0.85 ± 0.24^###^	0.94 ± 0.25^###^	1.2 ± 0.31^#^
CK (mmol/L)	334 ± 193	345 ± 268	197 ± 129	331 ± 386	201 ± 95	280 ± 147
L/B (%)	3.5 ± 0.27	3.5 ± 0.27	3.6 ± 0.58	3.3 ± 0.40	3.2 ± 0.68	3.2 ± 0.79

## Data Availability

Data will be made available on request.
